# Supplementary material for: Microbe-Derived Antioxidants Protect IPEC-1 Cells from H2O2-Induced Oxidative Stress, Inflammation and Tight Junction Protein Disruption via Activating the Nrf2 Pathway to Inhibit the ROS/NLRP3/IL-1β Signaling Pathway
Source: Antioxidants (Basel). 2024 Apr 27;13(5):533. doi: 10.3390/antiox13050533 (PMC11117695; doi:10.3390/antiox13050533)
Supplement: Supplementary file 1 [file antioxidants-13-00533-s001.zip › antioxidants-2946573-supplementary.pdf]

Table S1. Primers used in current study.

Gene	Forward primer (5'-3')	Reverse primer (5'-3')	Accession number
NLRP3	CAGCACGAACCAGAATCTCA	AGCAGCAGTGTGATGTGAGG	NM_001256770.2
ASC	ACAACAAACCAGCACTGCAC	CTGCCTGGTACTGCTCTTCC	AB873106.1
Casepase-1	TTTGAAGGACAAACCCAAGG	TGGGCTTTCTTAATGGCATC	NM_214162.1
TNF-α	CGCTCTTCTGCCTACTGCACTTC	CTGTCCCTCGGCTTTGACATT	JF831365.1
IL-6	CCAGGAACCCAGCTATGAAC	CTGCACAGCCTCGACATT	AF518322.1
IL-18	CTGCTGAACCGGAAGACAAT	CTCAAACACGGCTTGATGTC	AF191088.1
IL-1β	CCAAAGAGGGACATGGAGAA	TTATATCTTGGCGGCCTTTG	NM_001302388.2
Nrf2	CACCACCTCAGGGTAATA	GCGGCTTGAATGTTTGTC	XM_021075133.1
NQO1	GTGGAAGCCGCAGACCTTGTG	CATGGCAGCGTATGTGTAAGCAAAC	NM_001159613.1
HO-1	AGGCTGAGAATGCCGAGTTC	TGTGGTACAAGGACGCCATC	NM_001004027.1
ZO-1	ACCCACCAAACCCACCAA	CCATCTCTTGCCAAACTATC	XM_013993251.1
Occludin	GCTGGAGGAAGACTGGAT	ATCCGCAGATCCCTTAAC	NM_001163647.2
β-actin	CTGCGGCATCCACGAAACT	AGGGCCGTGATCTCCTTCTG	ON164673.1

Table S2. siRNAs fragment sequence used in current study.

Gene	Forward primer (5'-3')	Reverse primer (5'-3')
si NLRP3	GCCUUAAGUUGUGUGAAAUTT	AUUUCACACAACUUAAGGCTT
si Nrf2	GCCCAUUGAUCUCUCUGAUTT	AUCAGAGAGAUCAAUGGGCTT

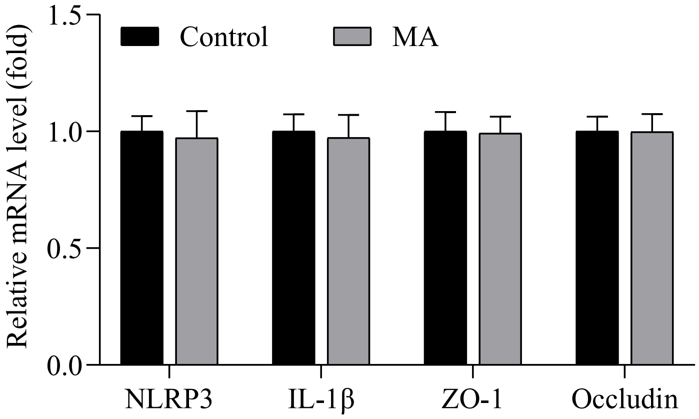

Figure S1 Effect of MA treatment on mRNA expression of NLRP3, IL-1β, ZO-1 and Occludin. IPEC-1 cells (1 × 10^5 cells/mL, 2 mL) were inoculated in 6-well plates, and cultured at 37°C for 24 h. Two groups were set up:Control group (cells only) and MA group (100 μg/mL MA treatment for 12 h). Cells were collected to extract RNA for qPCR detection.
